# Successful pregnancy outcome in Swyer-James-Macleod syndrome

**DOI:** 10.4103/1817-1737.37976

**Published:** 2008

**Authors:** Seema Chopra, Neelam Aggarwal, Vanita Suri, Meenakshi R. Thami

**Affiliations:** *Department of Gynecology and Obstetrics, Post Graduate Institute of Medical Education and Research, Chandigarh - 160012, India*

**Keywords:** Bronchiectasis, hyperlucent lung, Swyer-James syndrome

## Abstract

Swyer-James-MacLeod (SJM) syndrome is a chronic, progressive lung disease as a result of infection and bronchial obstruction that ultimately leads to emphysema. It is associated with chronic cough, sputum production and recurrent chest infections and is occasionally seen in women of reproductive age. The radiological finding of unilateral hyperlucent lung is considered synonymous with the disease entity.

## Introduction

Swyer-James-MacLeod syndrome is considered to be an acquired disease secondary to viral bronchiolitis and pneumonitis in childhood.[1] The resulting bronchiectasis predisposes to recurrent bronchopulmonary infections. As the disease usually manifests in infancy or childhood, it is not frequently seen in obstetric practice. In the literature, only a few case reports are available documenting various pregnancy outcomes in association with this syndrome. Reported here is successful pregnancy outcome in a patient with MacLeod syndrome.

## Case Report

A 30-year-old primigravida, diagnosed as a case of Macleod syndrome for the past 12 years, was supervised in our antenatal clinic. She had received antitubercular drugs for pulmonary tuberculosis at 8 years of age; and later on, intercostal chest tube drainage for empyema thoracis at her native place. She had recurrent episodes of fever, breathlessness and cough when she had presented in the Department of Pulmonary Medicine at our institute. Chest X-ray revealed old lesions of pulmonary Koch's with cicatrisation collapse. Computed tomographic scan showed decreased attenuation of right lung and basal segment of left lung, diminished right hilar vessels and bronchiectasis, consistent with the Swyer-James syndrome.

She had regular follow-up in our clinic. General physical examination showed tracheal deviation to right side. Chest auscultation revealed diffuse crepitations in right lung fields. Spirometry revealed moderate obstruction with FEV1 (forced expiratory volume in one second) IOL, FEV1 / FVC (forced vital capacity) 69%. Maternal and fetal surveillance was uneventful. She was normotensive till 34 weeks of gestation, when she developed severe preeclampsia and features of imminent eclampsia, for which labor was induced with oxytocin. Intrapartum arterial blood gas monitoring on room air showed PO_2_ 77 mmHg, PCO_2_ 28 mmHg, pH of 7.40, O_2_ saturation 97.5%. She developed non-reassuring fetal heart rate patterns, and a live-born male baby weighing 1,830 g with normal Apgar score was delivered by emergency cesarean section under spinal anesthesia. Broad-spectrum antibiotics were given for surgical intervention, as well as pulmonary infection prophylaxis. Intraoperative and postoperative period was uneventful, and both the mother and the child were discharged in a satisfactory condition.

## Discussion

The syndrome was first described by Swyer and James in 1953. Macleod later reviewed the clinical, radiological and bronchographic features in 1954. Roentgenographic hypertransradiancy of one lung and its decreasing size are the main features. Hypoplasia of lung vessels and emphysema secondary to chronic inflammation of bronchioles aid in the diagnosis.[2] Computed tomographic scan shows emphysematous changes, and perfusion scintigraphy demonstrates absence of blood flow in the affected lung.

SJM syndrome is a result of unilateral post-infectious bronchiolitis obliterans, usually due to mycoplasma or viral lung infections, in infancy and early childhood.[1,3] The suppurative pulmonary infection leads to destruction of bronchial epithelium, muscle and elastic tissue. The resulting loss of ciliary action and therefore impaired bronchial drainage predisposes to secondary infections.[4] The clinical consequences include shortness of breath, atelectasis, bronchiectasis and unilateral hyperlucent lung due to loss of pulmonary vascular structure and alveolar over-distension.[1] Physical examination demonstrates crackles in almost all and wheezes in some patients.

Swyer-James-MacLeod syndrome can also occur in association with tuberculosis (as in our case), foreign body obstructing the bronchus, congenital disorders such as situs inversus.[4] In a series of 40 patients, differential diagnosis of chronic hyperlucent lung included Swyer-James syndrome (45%), localized emphysema (20%), congenital hypoplastic pulmonary artery (10%), bronchial carcinoma (7.5%), postradiotherapy (5%) and benign intrabronchial neoplasm (2.5%), with most extensive pulmonary vasculature damage in the Swyer-James syndrome.[5]

‘Recurrent pulmonary infections’ is the usual course of the disease process. Therefore, treatment includes early control of lung infections with antibiotics.[3,6] Surgical treatment is indicated in cases of chronic infection of the damaged lung.[2] In the literature, pneumonectomy[1] or occlusion of the main bronchus by its resection from the affected lung without pneumonectomy[2] are described as treatment modalities.

Although the pathological process usually manifests at a young age, the clinical incidence in pregnancy is low and is stated to be 1:2500.[4] In a review of 44 pregnancies in 21 patients, pregnancy was not adversely affected because of the underlying lung pathology.[4] Similarly, none of the 3 patients of this syndrome had worsening of symptoms during pregnancy and fetal growth was unaffected, as reported by Howie.[7] However, maternal hypoxemia leading to growth restriction and intrauterine fetal death in a pregnant patient with bronchiectasis and prior lobectomy is also reported.[8] Similarly, Thaler[6] reported an obstetric patient with bronchiectasis who had repeated episodes of fever and pulmonary infections with deterioration of arterial blood gases, for which she needed antibiotics and oxygen. Pregnancy termination at 33 weeks ended in a small-for-date baby weighing 1,650 g. The occurrence of preeclampsia in our patient can be attributed to maternal hypoxia, as is also suggested in the studies[9,10] correlating hypoxia with preeclampsia. Preeclampsia and intra uterine growth retardation are known to occur in association with placental hypoxia. Hypoxia-inducible factor may facilitate placental oxygen transfer by increasing angiogenesis in placenta, maternal erythropoiesis and vascular endothelial growth factor production.[9]

Increased severity of asthma leading to maternal hypoxia was correlated with increased risk of preeclampsia compared to asymptomatic women with diagnosis of asthma (odds ratio 3.36).[10] Our patient fortunately did not have any feature of respiratory infections during her pregnancy. She stood the labor and surgery well, without any deterioration of blood gases or respiratory failure and had a good perinatal outcome.

Our report of successful outcome of pregnancy in a patient of Swyer-James syndrome - Macleod syndrome is consistent with good outcome reported in literature. The obstetric management entails careful antepartum maternal and fetal surveillance in terms of pulmonary infections and use of appropriate antibiotics, pulmonary function tests, early detection of growth restriction and timely intervention to deliver a healthy baby.
